# Clinical Features and Treatment of Patients Infected With SARS-CoV-2 Omicron Variant While Hospitalized Due to Stroke: A Single Center Study in Japan

**DOI:** 10.7759/cureus.54760

**Published:** 2024-02-23

**Authors:** Kazuki Miyazaki, Hiroshi Kanno, Sachiko Yamada, Yuuki Sagehashi, Shutaro Matsumoto, Satoru Takahashi, Yongson Kim, Keiko Namiki, Satoshi Fujii

**Affiliations:** 1 Department of Neurosugery, Asahi Hospital, Tokyo, JPN; 2 Department of Neurosurgery, Asahi Hospital, Tokyo, JPN; 3 Department of Pharmacy, Asahi Hospital, Tokyo, JPN

**Keywords:** therapeutic strategy, coagulopathy, modified rankin scale, sars-cov-2 omicron variant, covid-19, stroke

## Abstract

Background and objective: In December 2019, COVID-19 spread rapidly across the globe. Throughout the pandemic, SARS-CoV-2 repeatedly mutated, transitioning from the alpha variant to the omicron variant. The severity and mortality of COVID-19 have been linked to age, sex, and the presence of underlying diseases (respiratory, cerebrovascular, cardiovascular, metabolic, and immune diseases, as well as cancer). The clinical features of patients infected with COVID-19 following a stroke, however, are fully unknown. Therefore, it is significant to explore the appropriate treatment for these patients based on their clinical features.

Methods: Of the 6175 patients who visited Asahi Hospital (Tokyo, Japan) between November 2022 and February 2023, 206 were admitted. Of these 206 patients, the 44 that contracted COVID-19 while hospitalized for strokes were retrospectively analyzed.

Results: Six (13.6%) of these patients died; four expired due to coagulopathy associated with ischemic heart failure and recurrent ischemic cerebrovascular disease. The mean D-dimer level increased to 3.53 in the deceased patients, while it was 1.64 in all patients. The platelet count was low in three of the deceased patients, while it was high in two patients. The severity of COVID-19 was significantly correlated with a high modified Rankin Scale (mRS) score and a high National Institute of Health Stroke Scale (NIHSS) score. The timing of vaccination is inversely correlated with COVID-19 severity.

Conclusion: Patients with COVID-19 after a stroke have high mortality rates due to coagulopathy. Stroke patients with high mRS scores and high NIHSS scores are more likely to develop severe COVID-19. Anticoagulant therapy should be administered to COVID-19 patients with high mRS scores following a stroke.

## Introduction

The SARS-CoV-2 virus causes the infectious disease COVID-19, which spread rapidly worldwide since December 2019, resulting in an unprecedented pandemic. Throughout the pandemic, SARS-CoV-2 underwent successive mutations, transitioning from the alpha to the omicron variant [1]. The SARS-CoV-2 belongs to the betacoronavirus family and is a single-stranded RNA virus with an envelope [2]. Historically, human coronaviruses, including human coronavirus (hCoV)-OC43, hCoV-HKU, and hCoV-229E, were known to induce mild cold-like symptoms only [3]. The COVID-19 clinical guidelines of the Ministry of Health, Labor, and Welfare in Japan state that the severity and mortality classification of COVID-19 and its complications are related to age, species, and underlying diseases, including stroke [4].

A history of stroke increases the likelihood of developing severe complications from COVID-19 by 2.55 times [5]. Patients with COVID-19 who have a history of stroke are more likely to develop severe respiratory disease, have lower discharge rates, and have higher mortality rates [6]. By contrast, the occurrence of a stroke following COVID-19 is more common in elderly patients with hypertension, diabetes, or a history of stroke [7]. The incidence of acute stroke following COVID-19 is 4.6% [7], and the pooled prevalence of ischemic stroke in COVID-19 is 2% [8]. Ischemic stroke commonly occurs approximately two weeks after the onset of COVID-19 symptoms [9].

The clinical features and therapeutic strategies for patients who contracted COVID-19 during hospitalization for strokes have yet to be reported. Interestingly, the clinical features of patients infected with COVID-19 after a stroke differ from those of patients who have a stroke after COVID-19. Furthermore, the features and mechanisms of coagulopathy associated with COVID-19 after a stroke have not been completely clarified. Also, the risk of severe COVID-19 following a stroke remains unknown. Therefore, based on the clinical features, exploring the appropriate treatment for those patients is significant.

The population with COVID-19 and patients who develop a stroke after COVID-19 are not usually prescribed anticoagulant drugs, while patients hospitalized after a stroke have always been prescribed anticoagulant drugs. Nevertheless, coagulopathy occurs in COVID-19 infections following a stroke. Therefore, this study aims to present the clinical features of patients who contracted COVID-19 while hospitalized due to strokes and examine whether anticoagulant addition is necessary. This study also proposes appropriate therapeutic strategies for COVID-19 after a stroke.

## Materials and methods

**Figure 1:**
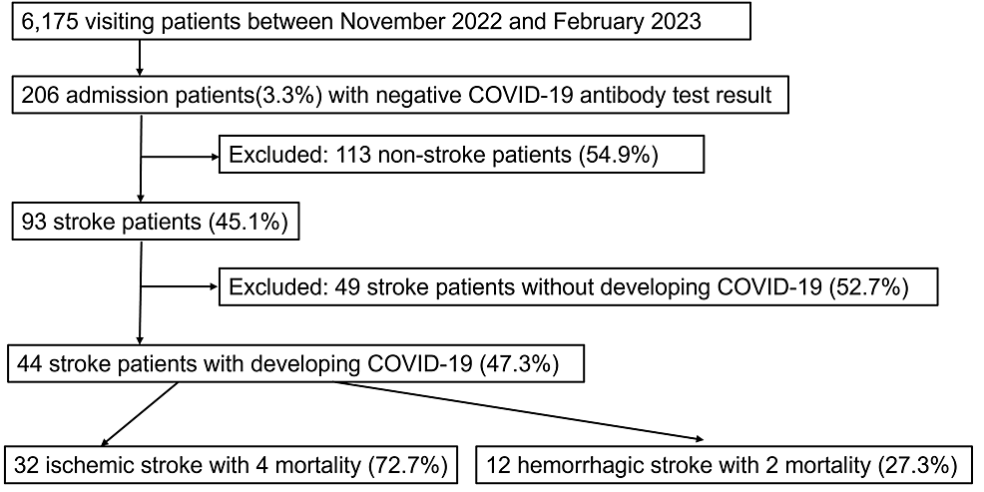
Flowchart depicting the process of identifying patients for this study

Of the 6175 patients who visited the Asahi Hospital (Tokyo, Japan) between November 2022 and February 2023, 206 patients with negative COVID-19 antibody test results were admitted to the hospital. Of these 206 patients, 93 were admitted due to a stroke, of which 44 were infected with COVID-19 (diagnosed by a COVID-19 antibody test or polymerase chain reaction (PCR) test) and retrospectively evaluated (Figure 1). The sample size was calculated from records of visiting patients and admission patients between November 2022 and February 2023. This study was approved by the Institutional Review Board of the Asahi Hospital (approval no. TAH2023-2). Written and informed consent for the publication of research details and clinical images was obtained from all patients.

The following variables were obtained: the number, sex, and age of patients infected with the SARS-CoV-2 omicron variant while being hospitalized to treat a stroke; stroke type and subclassification; symptoms; complications; drugs used for treating COVID-19; number of COVID-19 vaccine doses; median days from onset of stroke to infection with COVID-19; modified Rankin Scale (mRS) score [10] at COVID-19 onset; National Institute of Health Stroke Scale (NIHSS) score at COVID-19 onset; the severity of COVID-19 per the severity classification of the COVID-19 clinical guidelines version 9.0 of the Ministry of Health, Labor, and Welfare in Japan [11]; and images of a representative patient who expired. According to the severity classification of the COVID-19 clinical guidelines version 9.0 of the Ministry of Health, Labor, and Welfare in Japan, a saturation of percutaneous oxygen (SpO2) >93% and cough only without pneumonia is considered mild; SpO2 <93%, pneumonia with dyspnea, and the need for O2 supply is considered moderate; it is considered severe when management under ICU is needed. Occasionally, artificial ventilation is applied.

Furthermore, the correlation between the severity of COVID-19 and the surveyed values was analyzed. The surveyed values were as follows: age (≥80 years or ≤80 years), sex (male or female), period from stroke to COVID-19 onset (≥50 days or ≤50 days), number of vaccination doses, period from last vaccination to stroke onset, mRS at COVID-19 onset, NIHSS at COVID-19 onset, stroke type (ischemic or hemorrhagic), and subtype of ischemic stroke (atherosclerotic, cardiogenic, or lacunar) [12]. Platelet count (×104/μL), D-dimer (μg/mL), and prothrombin time-international normalized ratio (PT-INR) were the laboratory parameters used for assessing coagulopathy.

Statistical analysis

The Statcel4 statistical software (OMS Publishing Inc., Tokorozawa, Japan) was utilized. The data were analyzed using Welch’s t-test, chi-square test for independence, Mann-Whitney U-test, or single-factor analysis of variance, as appropriate. A p-value of <0.05 was considered significant.

## Results

Characteristics of patients infected with COVID-19 following a stroke

A total of 44 stroke patients admitted to our hospital between November 2022 and February 2023 were infected by the SARS-CoV-2 omicron variant. The genetic variant of SARS-CoV-2 identified was a spike protein-mutated type L452R, omicron BA.5.2.6. The type of SARS-CoV-2 omicron variant was diagnosed with the whole genome sequence of SARS-CoV-2. All patients who were diagnosed with COVID-19 after suffering a stroke in this study were administered the BNT162b2 vaccine (Pfizer-BioNTech). The ages of the 44 patients ranged from 53 to 95 years, with a median age of 81 years. The study population comprised 30 men and 14 women. As for stroke type, 32 patients had ischemic stroke (eight cardiogenic, 14 atherosclerotic, and 10 lacunar), while 14 patients had hemorrhagic stroke, all of whom were hypertensive. The NIHSS scores at COVID-19 onset ranged from 1 to 30, with a mean of 13.5. The mRS scores at COVID-19 onset were mRS0, 1; mRS1, 1; mRS2, 2; mRS3, 15; mRS4, 13; and mRS5, 10, with a median score of 4. The underlying diseases included hypertension (n = 14), diabetes mellitus (n = 9), arterial fibrillation (n = 7), chronic kidney disease (n = 2), bronchial asthma (n = 2), and cancer (n = 2). The primary complications associated with COVID-19 were acute myocardial infarction (n = 3), cerebral infarction (n = 1), and severe leukopenia (n = 1). The distribution of patients by COVID-19 severity was as follows: mild in 33; moderate in four; severe in seven. Six of the seven patients with severe COVID-19 died. The mortality rate of post-stroke patients with COVID-19 was 13.6%. The distribution of the number of COVID-19 vaccination doses was as follows: none (n = 2); one (n = 4); two (n = 12); three (n = 19); four (n = 6); and five (n = 1). Hospitalized stroke patients were easily infected with COVID-19, regardless of vaccination time. The symptoms included fever (n = 38), cough (n = 31), pneumonia (n = 12), heart failure (n = 6), and leukopenia (n = 1). The period from stroke to COVID-19 ranged from three to 132 days (median: 46.5 days). The modalities used for treating COVID-19 included molnupiravir monotherapy (n = 37); combination therapy with molnupiravir and dexamethasone (n = 3); and molnupiravir, dexamethasone, and anticoagulant drugs (n = 4) (Table 1).

**Table 1:** Characteristics of patients who contracted COVID-19 following a stroke ICH: Intracerebral hemorrhage; NIHSS: National Institute of Health Stroke Scale; mRS: Modified Rankin Scale

Characteristics		Value	Mortality
No. of patients		44 (100%)	6 (13.6%)
Age (in years)	Range	53 to 95	77 to 89
	Median	81	85
Sex	Male	30 (68.2%)	4 (13.3%)
	Female	14 (31.8%)	2 (14.3%)
Stroke type	Ischemic stroke	32 (72.7%)	4 (66.7%)
	Cardiogenic	8 (18.2%)	1 (16.7%)
	Atherosclerotic	14 (31.8%)	3 (50%)
	Lacunar	10 (22.7%)	0 (0%)
	Hemorrhagic stroke	12 (27.3%)	2 (33.3%)
	Hypertensive ICH	12 (27.3%)	2 (33.3%)
NIHSS score at COVID-19 onset	Range	1 to 30	14 to 30
	Mean	13.5	23.7
mRS score at COVID-19 onset	mRS0	1 (2.2%)	0 (0%)
	mRS1	1 (2.2%)	0 (0%)
	mRS2	2 (4.5%)	0 (0%)
	mRS3	15 (34.1%)	0 (0%)
	mRS4	13 (29.5%)	1 (16.7%)
	mRS5	10 (22.7%)	5 (83.3%)
Underlying disease	Hypertension	14 (31.8%)	1 (16.7%)
	Diabetes mellitus	9 (20.5%)	1 (16.7%)
	Arterial fibrillation	7 (15.9%)	1 (16.7%)
	Chronic kidney disease	2 (4.5%)	2 (33.3%)
	Bronchial asthma	2 (4.5%)	1 (16.7%)
	Cancer	2 (4.5%)	0 (0%)
Severe complication	Acute myocardial infarction	3 (6.8%)	3 (50%)
	Cerebral infarction	1 (2.3%)	1 (16.7%)
	Heart failure	6 (13.6%)	1 (16.7%)
	Leukopenia	1 (2.3%)	1 (16.7%)
Severity of COVID-19	Mild	33 (75%)	NA/(-)
	Medium	4 (9.1%)	NA/(-)
	Severe	7 (15.9%)	NA/(-)
No. of COVID-19 vaccination doses	0	2 (4.5%)	2 (33.3%)
	1	4 (9.1%)	2 (33.3%)
	2	12 (27.3%)	1 (16.7%)
	3	19 (43.2%)	1 (16.7%)
	4	6 (13.6%)	0 (0%)
	5	1 (2.3%)	0 (0%)
Symptoms	Fever	38 (86.4%)	6 (100%)
	Cough	31 (70.5%)	6 (100%)
	Pneumonia	12 (27.3%)	6 (100%)
Time from stroke to COVID-19 onset (in days)	Range	3 to 132	11 to 132
	Median	46.5	59
Time from last vaccination to stroke (in days)	Range	15 to 320	27 to 320
	Median	75	90
Therapy for COVID-19	Molnupiravir only	37 (84.1%)	6 (100%)
	Molnupiravir and dexamethasone	3 (6.8%)	0 (0%)
	Molnupiravir, dexamethasone, and anticoagulant	4 (9.1%)	0 (0%)

Laboratory data on platelet count, D-dimer levels, and PT-INR

The parameters considered in the analysis were age (<80 or >80 years), sex (male or female), stroke type (ischemic or hemorrhagic), mRS score (0-3 or 4-5), number of vaccination doses (0-2 or 3-5), period from stroke to COVID-19 (<50 or >50 days), and severity of COVID-19 (mild, moderate, or fatal). In terms of COVID-19 severity, the D-dimer levels in patients with mild COVID-19 were significantly lower than those in patients with moderate to fatal COVID-19 (p<0.01). The PT-INR in ischemic stroke was significantly different from that in hemorrhagic stroke (p<0.05). Similarly, the PT-INR in patients with a duration of <50 days from stroke to COVID-19 onset or mild COVID-19 was significantly lower than in patients with a duration of >50 days (p<0.05) from stroke to COVID-19 onset or moderate to fatal COVID-19. In addition, the PT-INR in patients with cardiogenic ischemic stroke was significantly greater than in patients with other subtypes of ischemic stroke (p<0.05). The other parameters were not significant. Three of the six deceased patients demonstrated a low platelet count, while two exhibited a high platelet count (Table 2).

**Table 2:** Characteristics of the six deceased stroke patients with COVID-19 mRS: Modified Rankin Scale; CI: Cerebral infarction; ICH: Intracerebral hemorrhage: AF: Arterial fibrillation; DM: Diabetes mellitus; CKD: Chronic kidney disease; CHF: Chronic heart failure; HT: Hypertension; AMI: Acute myocardial infarction; IHF: Ischemic heart failure; PT-INR: Prothrombin time-international normalized ratio

Age (in years)	Sex	mRS score	NIHSS score	No. of vaccination doses	Stroke type	Subtype	Underlying disease	Days from stroke to COVID-19 onset	Symptoms of COVID-19	Therapy for COVID-19	COVID-19-associated disease	Platelet count (×10^4^/μL)	D-dimer (μg/mL)	PT-INR
85	M	5	25	0	CI	Cardiogenic	AF, DM	55	Fever, cough, pneumonia, pleural effusion	Molnupiravir	AMI	12.5	2.33	1.8
89	M	5	26	1	CI	Atherosclerotic	CKD, CHF	79	Fever, cough, pneumonia	Molnupiravir	ARF, AMI	37.3	7.13	1.03
77	M	4	14	3	CI	Atherosclerotic	CKD	11	Fever, cough, pneumonia, pleural effusion	Molnupiravir	CI (1), CI (2), AMI	25.5	3.49	1.25
85	M	5	30	1	ICH	Hypertensive	HT	132	Fever, cough, pneumonia,	Molnupiravir	IHF, Leukopenia	5.8	1.99	1
82	F	5	24	0	ICH	Hypertensive	Asthma	52	Fever, cough, pneumonia	Molnupiravir	None	35.2	1.15	1
85	F	5	23	0	CI	Atherosclerotic	None	61	Fever, cough, pneumonia	Molnupiravir	None	8.4	5.09	1.5

Mortality in patients diagnosed with COVID-19 after a stroke 

**Figure 2:**
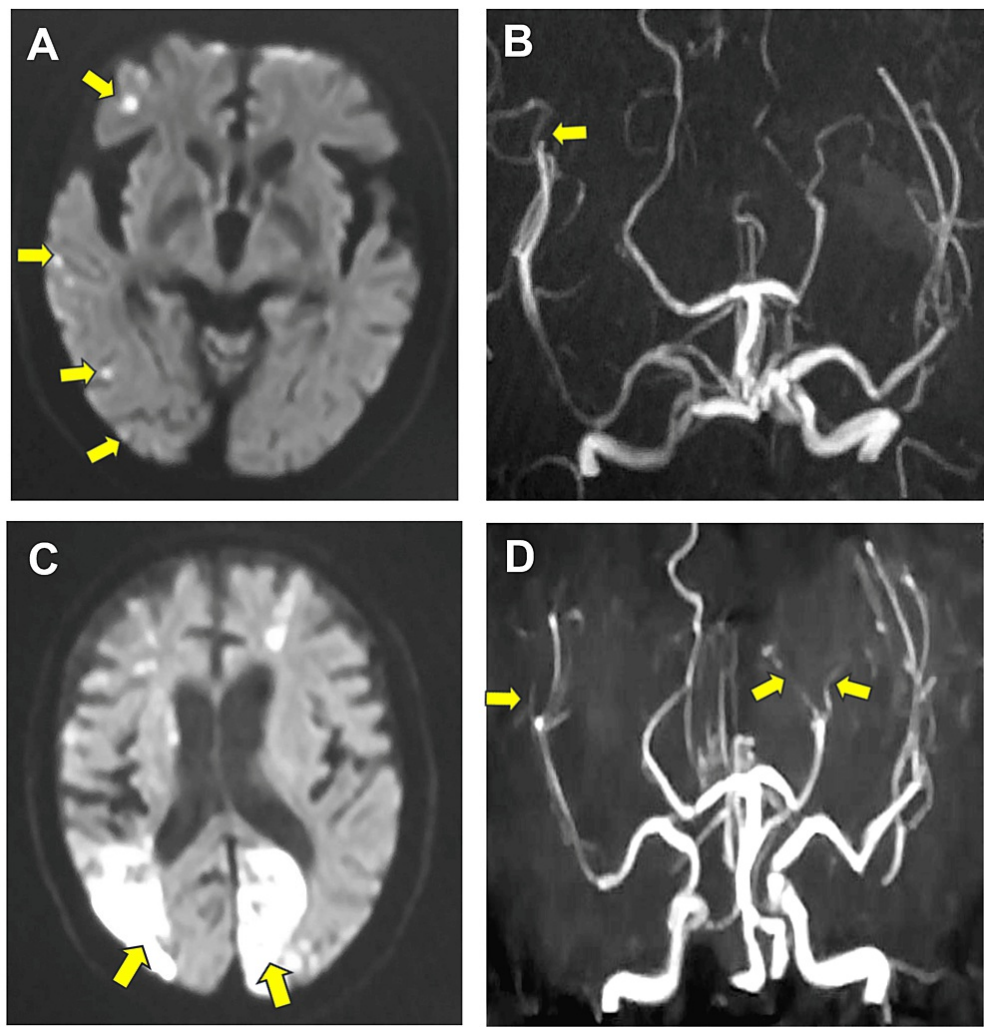
MRI of a deceased stroke patient who contracted COVID-19 during hospitalization A: Diffusion-weighted image (DWI) at the time of admission shows cerebral infarction. Scattered high-intensity spots are observed in the right cerebral hemisphere (yellow arrows). B: An MR angiography at the time of admission reveals cerebral infarction. A large vessel occlusion is noted (yellow arrow). C: DWI performed within 20 days after COVID-19 onset shows high-intensity areas in the left occipital lobe and right parietal lobe (yellow arrows). D: The left posterior cerebral artery and right middle cerebral artery branches are occluded. Cryptogenic large vessel occlusions were identified in bilateral hemispheres after COVID-19 infection (yellow arrows).

Among the stroke patients with COVID-19, six died (13.6%). The average age of patients who died was 83.8 years (median: 85 years). Among the patients, four were men, and two were women. The mRS score at the time of stroke occurrence was 5 in five of the six patients, while the remaining patient had an mRS score of 4. The NIHSS score at the onset of COVID-19 ranged from 14 to 30, with a mean of 23.7. The average number of vaccine doses received was 1.2 (median: 1 dose). The causes of death were pneumonia (n = 3), myocardial infarction (n = 2), and cerebral infarction with myocardial infarction (n = 1). The stroke types were cerebral infarction in four patients (three atherosclerotic and one cardiogenic) and intracerebral hemorrhage in two. The underlying diseases included chronic kidney disease (n = 2), diabetes mellitus (n = 1), bronchial asthma (n = 1), chronic heart failure (n = 1), hypertension (n = 1), and arterial fibrillation (n = 1). The median duration from stroke to COVID-19 onset was 70 days. The COVID-19 symptoms, including fever, cough, and pneumonia, were present in all patients, with two patients showing pulmonary edema. The critical complications associated with COVID-19 included myocardial infarction (n = 3), acute kidney injury (n = 1), two instances of large vessel occlusion (LVO)-type cerebral infarction (n = 1), and severe leukopenia (<500/mm3) (n = 1) [13]. The treatment provided to all COVID-19 patients consisted exclusively of molnupiravir. One of the deceased patients experienced a significant decline in mRS score after contracting COVID-19 on the 11th day of hospitalization for cerebral infarction, leading to LVO-type cerebral infarctions within 10 and 18 days after disease onset. Table 3 presents the coagulation data of patients infected with COVID-19 while being hospitalized for strokes. Figure 2 is the MRI scan of one of the deceased patients who died of recurrent cerebral infarction and myocardial infarction following the onset of COVID-19 during hospitalization for ischemic stroke.

**Table 3:** Laboratory data of coagulation in patients who contracted COVID-19 following a stroke PT-INR: Prothrombin time-international normalized ratio, mRS: Modified Rankin Scale

Parameters	No. of patients, n (%)	Platelet count (×10^4^/μL, mean±SD)	D-dimer (μg/mL,mean±SD)	PT-INR (mean±SD)
All patients	44 (100%)	22.7±7.2	1.64±1.19	1.06±0.25
Age (in years)				
<80	16 (36.4%)	23.4±10.8	1.36±0.60	1.03±0.13
>80	28 (63.6%)	22.2±8.0	1.72±1.14	1.08±0.22
Sex				
Male	30 (68.2%)	22.9±7.3	1.71±1.65	1.06±0.19
Female	14 (31.8%)	22.3±7.2	1.89 ± 1.66	1.04± 0.16
Stroke type				
Ischemic	32 (72.7%)	22.4±6.9	1.95 ± 1.84	1.09±0.22
Ischemic stroke subtype				
Atherosclerotic	14 (31.8%)	24.1±7.2	2.74 ± 2.20	1.08±0.15
Cardiogenic	8 (18.2%)	18.6±5.6	1.39 ± 0.87	1.24±0.34
Lacunar	10 (22.7%)	23.2±6.8	0.71 ± 0.62	0.99±0.07
Hemorrhagic	12 (27.3)	23.3±7.8	1.22 ± 0.68	0.98±0.19
mRS score				
0 to 3	21 (47.7%)	23.5±6.5	1.23 ± 0.84	1.01±0.24
4 to 5	23 (52.3%)	22.0±7.9	1.93 ± 1.73	1.09±0.25
No. of vaccination doses				
0 to 2	18 (40.9%)	23.6±8.4	2.53 ± 1.97	1.13±0.26
3 to 5	26 (59.1%)	22.0±6.4	1.58 ± 0.89	1.01±0.23
Period from stroke to COVID-19				
<50 days	22 (50%)	22.7±6.3	1.42 ± 0.91	1.00±0.09
>50 days	22 (50%)	22.6±8.2	1.99 ± 0.34	1.23±0.23
Severity of COVID-19				
Mild	36 (81.8%)	23.1±5.8	1.00 ± 0.63	0.86±0.20
Moderate to severe	8 (18.2%)	23.4±10.7	2.71 ± 1.78	1.21±0.31

Association between the variables of the patients

The mRS scores and COVID-19 severity were significantly correlated (p = 0.044). These results demonstrate that stroke patients with higher mRS scores experience more severe COVID-19 than those with lower mRS scores. Significant differences were identified between the mRS of deceased stroke patients who contracted COVID-19 and the mRS of survivors (p = 0.015). In addition, the NIHSS scores were significantly correlated with the severity of COVID-19 (p = 0.0088). No significant difference was observed in the risk of COVID-19 severity between patients with ischemic strokes and those with hemorrhagic strokes. There was no significant difference found between ischemic stroke subtypes. Moreover, no significant difference was observed in the risk of COVID-19 severity between patients aged ≥80 years and those aged ≤80 years (p = 0.17). Similarly, no significant difference was found in COVID-19 severity between sexes (p = 0.96). The number of days between stroke onset and COVID-19 manifestation did not differ in terms of COVID-19 severity (p = 0.29). A significant association was found between the number of COVID-19 vaccination doses and disease severity (p = 0.016), with fewer numbers correlating to a higher risk of disease severity. On the other hand, no significant difference was found between the period from the last vaccination to stroke onset and disease severity. Higher mortality rates were observed in patients with chronic kidney disease and bronchial asthma (Table 4).

**Table 4:** Association between the characteristics of patients and the severity of COVID-19 A p-value of <0.05 is considered statistically significant. mRS: Modified Rankin Scale; NIHSS: National Institute of Health Stroke Scale

Characteristics	Severity of COVID-19				p-value
	Mild	Moderate	Severe	Deceased	
Age (in years)					p = 0.17
<80	15 (34.1%)	0 (0%)	0 (0%)	1 (2.2%)	
>80	18 (40.9%)	3 (6.8%)	2 (4.5%)	5 (11.4%)	
Sex					p = 0.96
Male	23 (76.7%)	2 (6.7%)	1 (3.3%)	4 (13.3%)	
Female	10 (71.4%)	1 (7.1%)	1 (7.1%)	2 (14.3%)	
Stroke type					p = 0.82
Ischemic	24 (75%)	2 (6.3%)	2 (6.3%)	4 (12.5%)	
Subtype of ischemic stroke					p = 0.6
Atherosclerotic	9 (64.3%)	1 (7.1%)	1 (7.1%)	3 (21.4%)	
Cardiogenic	6 (75%)	1 (12.5%)	0 (0%)	1 (12.5%)	
Lacunar	9 (90%)	0 (0%)	1 (10%)	0 (0%)	
Hemorrhagic stroke	9 (75%)	1 (8.3%)	0 (0%)	2 (16.7%)	
NIHSS score	33 (75.0%)	3 (6.8%)	2 (4.5.%)	6 (13.6%)	p = 0.0088
mRS score					p = 0.005
mRS0	1 (2.2%)	0 (0%)	0 (0%)	0 (0%)	
mRS1	1 (2.2%)	0 (0%)	0 (0%)	0 (0%)	
mRS2	1 (2.2%)	0 (0%)	0 (0%)	0 (0%)	
mRS3	17 (38.6%)	1 (2.2%)	0 (0%)	0 (0%)	
mRS4	11 (25.0%)	1 (2.2%)	0 (0%)	1 (2.2%)	
mRS5	2 (4.5%)	1 (2.2%)	2 (4.5%)	5 (11.4%)	
No. of vaccination doses					p = 0.016
0	0 (0%)	0 (0%)	0 (0%)	2 (4.5%)	
1	1 (2.2%)	0 (0%)	1 (2.2%)	2 (4.5%)	
2	10 (22.7%)	1 (2.2%)	0 (0%)	1 (2.2%)	
3	15 (34.1%)	2 (4.5%)	1 (2.2%)	1 (2.2%)	
4	6 (13.6%)	0 (0%)	0 (0%)	0 (0%)	
5	1 (2.2%)	0 (0%)	0 (0%)	0 (0%)	
Time from stroke to COVID-19 onset					p = 0.21
<50 days	19 (43.2%)	1 (2.2%)	1 (4.5%)	1 (4.5%)	
>50 days	14 (31.8.%)	2 (4.5%%)	1 (4.5%)	5 (22.7%)	
Time from last vaccination to stroke					p = 0.80
<50 days	11 (25.0%)	0 (0%)	0 (0%)	2 (4.5%)	
>50 days, <100 days	10 (22.7%)	1 (2.2%)	0 (0%)	2 (4.5%)	
>100 days	13 (29.5%)	2 (4.5%)	2 (4.5%)	2 (4.5%)	

## Discussion

In the present study, we retrospectively investigated the clinical features of 44 patients infected with the SARS-CoV-2 omicron variant during hospitalization following a stroke. The median age of the patients was 81 years, with an elevated risk associated with advanced age [14]. The risk of severe COVID-19 in patients aged ≤80 years was not significantly lower than that in patients aged ≥80 years (p = 0.17), possibly due to the small number of patients. Men are at high risk of developing COVID-19 [15]. However, this study did not reveal a significant difference in the risk of severe COVID-19 based on sex. In addition, no significant difference was noted in the risk of severe COVID-19 between the ischemic and hemorrhagic stroke groups. Also, no significant difference was observed between the subtypes of ischemic stroke; however, this result might be attributed to the small number of patients with ischemic stroke. In this study, a high mRS score and a high NIHSS score at the onset of COVID-19 significantly correlated with the risk of severe COVID-19, and a previous study showed a correlation between a high mRS score before a stroke and the severe risk of COVID-19 [16]. Underlying diseases, including renal disease, hypertension, diabetes mellitus, cardiovascular disease, and liver disease (except cerebrovascular disease), are among the risk factors for severe COVID-19. In addition, a history of smoking, current smoking, and obesity are associated with a high risk of mortality [15]. In this study, the number of COVID-19 vaccination doses was significantly and inversely correlated with the severity of COVID-19 (p = 0.016). On the other hand, there was no significant difference between the period from the last vaccination to stroke onset and disease severity. The median period from a stroke to COVID-19 onset was 46.5 days. The COVID-19 clusters in hospitals occurred during the median period. In addition, the major complications identified in this study were acute myocardial infarction, acute cerebral infarction of LVO type, acute heart failure, and leukopenia [13]. A previous study reported the cardiovascular diseases of acute heart failure and myocardial infarction [14]. Another study reported acute LVO in the brain [16]. 

**Figure 3:**
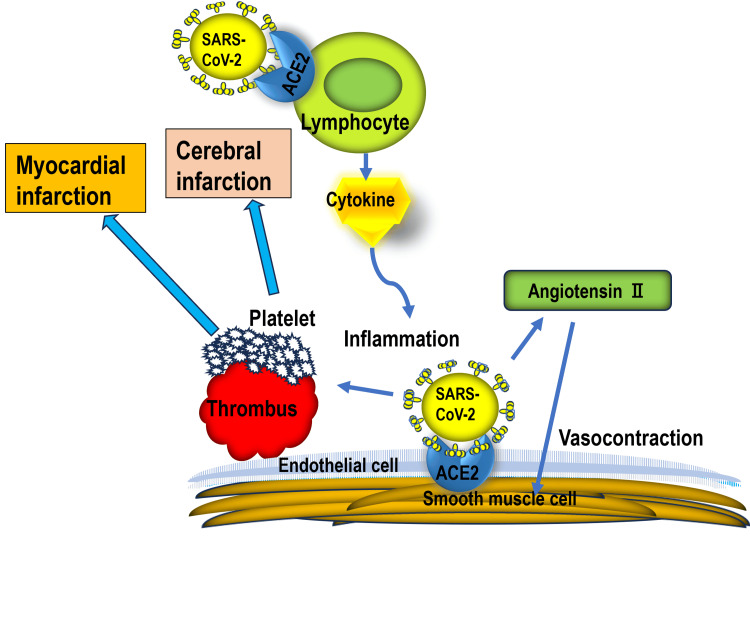
Mechanism of coagulopathy in COVID-19 As the SARS-CoV-2 virus enters a vessel, it binds ACE2 on a lymphocyte and releases cytokines. Additionally, it binds ACE2 on an endothelial cell and releases angiotensin Ⅱ, inducing vasoconstriction. Then, thrombus formation with platelet agglomeration is promoted, and cerebral or myocardial infarction occurs. ACE2: Angiotensin-converting enzyme 2 Diagram created by the authors.

The COVID-19 disease occurs when the S protein binds to the host cell receptor angiotensin-converting enzyme 2 (ACE2) and enters the cell. The S protein consists of a receptor-binding domain in the S1 region and a fusion-promoting S2 region. The latter facilitates fusion between the viral membrane and the host cell membrane. Angiotensin-converting enzyme 2 is highly expressed in the vascular endothelial cells. The severity of COVID-19 increases due to the infection of vascular endothelial cells, which subsequently leads to pulmonary vascular inflammation, thrombosis, angiogenesis, and other complications. Anticipating poor clinical outcomes in relation to COVID-19 is crucial. An increase in blood D-dimer levels correlates with disease severity. Vascular damage is associated with vasculitis and thrombosis in COVID-19 patients [17]. The characteristics of COVID-19 include respiratory syndrome and coagulopathy, as well as myocardial and cerebral infarctions (Figure 3). Large-vessel occlusion is a major type of cerebral infarction caused by COVID-19 [16]. Although the characteristics of COVID-19-associated coagulopathy (CAC) are distinct from those of other coagulopathies, the characteristic features of CAC partially overlap with the following four types: sepsis-induced disseminated intravascular coagulation, hemophagocytic lymphohistiocytosis, antiphospholipid syndrome, and thrombotic microangiopathy. A CAC is usually marked by elevated D-dimer and fibrinogen levels and initially shows minimal abnormalities in PT and platelet counts.

In this study, the D-dimer levels in patients with mild COVID-19 were significantly lower than those in patients with moderate to fatal COVID-19 (p<0.01). In addition, the PT-INR in patients with ischemic stroke (median duration from a stroke to COVID-19 onset of <50 days) and mild COVID-19 was significantly lower than in patients with hemorrhagic stroke (p<0.05) (median duration from a stroke to COVID-19 onset of >50 days, p<0.05) and moderate to fatal COVID-19 (p<0.05). Other parameters were not significant. Notably, three of the six deceased patients demonstrated a low platelet count, while two of the six deceased patients exhibited a high platelet count. The mechanism of thrombus formation in COVID-19 patients differs from that in healthy individuals. In healthy individuals, following the binding of angiotensin II to ACE2, angiotensin 1-7 stimulates the production of nitric oxide, leading to vasodilation and the inhibition of platelet aggregation. In COVID-19, however, SARS-CoV-2 binds to ACE2, and the angiotensin II levels are elevated, resulting in vasoconstriction and decreased blood flow. Then, the Willebrand factor (vWF) is released into the circulation, and platelet aggregation is promoted, resulting in thrombus formation. Furthermore, the interferon (IFN) pathway induces the expression of ACE2. Dysfunction of the IFN pathway in response to SARS-CoV-2 infection triggers a systemic cytokine storm. A cytokine storm can cause increased vascular permeability, hyperactivation of the coagulation-fibrinolysis system, disseminated intravascular coagulation (DIC), and respiratory failure [17]. It is also suggested that high plasminogen activator inhibitor type 1 (PAI-1) can lead to the development of blood clots due to a decrease in the ability to dissolve excess blood clots when they form [18]. The PAI-1 levels could independently predict disease severity and mortality rates for patients with COVID-19 [19].

Patients with COVID-19 are classified into mild, moderate, and severe groups according to symptoms, oxygen saturation, and underlying diseases [11]. Most patients with COVID-19 have a mild case, while a few develop a severe case, resulting in death. The severity and mortality of COVID-19 are related to age, sex, and underlying diseases (respiratory, cerebrovascular, cardiovascular, metabolic, and immune diseases, and cancer). The COVID-19 pandemic has significantly brought about a substantial transformation in stroke care, marked by a reduction in the number of patient visits, delayed medical consultations, and a decrease in the utilization of recombinant tissue plasminogen activator intravenous therapy and mechanical thrombectomy. A higher mortality rate has been reported in patients aged ≥70 years who have underlying conditions such as myocardial infarction, a history of stroke, arrhythmia, and high blood pressure [6]. A history of stroke increased the likelihood (2.55 times) of developing severe complications of COVID-19 [5]. Patients with COVID-19 who had a history of stroke were more likely to develop respiratory failure and had lower discharge and higher mortality rates. Another study showed that the incidence rate of stroke following hospitalization due to COVID-19 was 4.6% and was more common in elderly patients with hypertension, diabetes, or a history of stroke [7].

The pooled prevalence of ischemic stroke in patients with COVID-19 was 2%, and the pooled proportions of hypertension, hyperlipidemia, and diabetes in patients with COVID-19-related ischemic stroke were 66%, 48%, and 40%, respectively. According to the Trial of Org 10172 in acute stroke treatment (TOAST) classification, the trend of cryptogenic stroke subtypes was high among COVID-19 patients, with ischemic stroke obtaining a pooled proportion of 35% [8]. Ischemic stroke is more common approximately two weeks after the onset of COVID-19 symptoms [9]. Despite the administration of low-molecular-weight heparin as a prophylactic treatment, a thrombotic complication rate of 7.7% was observed. The prevalence of thrombosis in the venous system was notably high, and the incidence of stroke in the arterial system surpassed that of myocardial infarction. Thrombosis can also cause stroke and myocardial infarction. The occurrence of pulmonary embolism can lead to rapid respiratory deterioration. The current approach for treating patients with moderate to severe disease involves the combined use of antiviral and anti-inflammatory drugs. Anticoagulant drugs such as heparin are commonly administered to treat coagulopathy [20]. The COVID-19 disease is characterized by respiratory syndrome and coagulopathy, including myocardial and cerebral infarctions. An LVO is a major type of cerebral infarction in COVID-19 [16]. A previous study analyzed 9358 COVID-19 patients aged <50 years using multinational databases. Results showed that 33.2% of the patients had severe COVID-19 and required hospitalization. Ischemic stroke occurred in 64 patients (0.7%), with no significant differences according to sex [21]. Hypertension, diabetes, heart failure, nicotine dependence, obesity, chronic obstructive pulmonary disease, a history of stroke, and renal failure were significantly more common in patients who suffered a stroke. These patients had a mortality rate of 15.6%, which was significantly higher than in non-stroke patients (0.6%). Stroke associated with COVID-19 resulted in poor outcomes, particularly in younger individuals. Also, COVID-19 was an independent risk factor for LVO (p = 0.011) [22]. Patients with an ischemic stroke who had a history of respiratory infection within one week had a higher risk of LVO than those without such a history. This underscores the importance of further investigation into the specificity of LVO for COVID-19 [23].

Based on the laboratory data, elevated D-dimer levels were observed, and lupus anticoagulant and cardiolipin tests occasionally yielded positive results. A potential association is observed between lupus anticoagulant and anticardiolipin antibodies. A significant number of patients had poor clinical outcomes. These observations highlight the diverse and complex nature of stroke in patients with COVID-19. In a previous study examining the clinical types and outcomes, a substantial proportion of patients experienced cryptogenic stroke (65.6%), coupled with a high in-hospital mortality rate (63.6%) [24]. In another study that investigated 844 COVID-19 patients with stroke, 20 (2.4%) had ischemic stroke, while eight (0.9%) had hemorrhagic stroke. Patients with ischemic stroke had an average age of 64 years, with one patient (5%) aged <50 years. Among them, 95% had hypertension, while 60% had diabetes. The median time from the onset of COVID-19 symptoms to stroke diagnosis was 21 days. Of the patients who developed ischemic stroke, 40% had a cardioembolic type, 5% had a lacunar type, and 35% had a cryptogenic type [25]. In a separate study investigating the therapy of 174 patients with stroke following COVID-19, 34 (19.7%) patients received intravenous thrombolysis, while 21 (12.1%) patients underwent mechanical thrombectomy. A total of 48 deaths were reported (27.6%): 22 patients died due to COVID-19, while 26 died due to stroke. Of the 96 patients with available disability information, 49 (51%) exhibited severe disabilities at the time of discharge. The study also found that stroke occurred in 31 of 2132 (1.5%) patients with COVID-19 and in three of 1516 (0.2%) patients with influenza [26]. The incidence of stroke in patients with COVID-19 was approximately 7.5 times higher than in patients with influenza [27]. At the onset of stroke, SARS-CoV-2 enters the cells through the ACE2 receptors. The ACE2 is expressed in the lungs, heart, kidneys, and vascular endothelium, making it a potential target of the virus [28]. Damage to the heart increases the risk of thrombosis, and the associated endothelial damage can lead to vascular diseases, including stroke. Severe COVID-19 can lead to an uncontrolled immune response, resulting in a cytokine storm. Coagulation abnormalities such as elevated D-dimer levels may be a mechanism of stroke onset. And COVID-19 remained an independent risk factor for stroke (p = 0.001) (Figure 3) [29].

To date, various guidelines for managing COVID-19 have been proposed. Oral nirmatrelvir/ritonavir and molnupiravir are used in outpatients or mildly hospitalized patients, whereas intravenous remdesivir and sotrovimab are mainly used in hospitalized patients with moderate or severe disease. Dexamethasone has been administered in combination with these drugs. In this study, molnupiravir was administered to most of the patients. The patients who died also received molnupiravir, whereas those treated with remdesivir and dexamethasone survived. The risk-reduction ratio for molnupiravir was 30%, whereas for remdesivir it was 87% [30]. High-risk reduction drugs, such as remdesivir or sotrovimab, should be used in patients with a high risk of contracting COVID-19. The mortality rate of COVID-19 was reported to be >40% among hospitalized patients before the emergence of the omicron variant of SARS-CoV-2 [3]. Subsequently, this rate decreased to less than 10% among hospitalized patients, varying by country [31]. Among the 44 patients in this study, six (13.6%) died, which can be attributed to the influence of stroke in these patients. Severe complications, including myocardial infarction in three of six patients and recurrent cerebral infarction in one patient, were reported in those with fatal COVID-19, suggesting the involvement of COVID-19-associated coagulopathy. Additional administration of anticoagulants such as heparin, direct oral anticoagulants, and antiplatelet agents is considered necessary. Furthermore, all patients with fatal cases received molnupiravir alone as initial treatment for COVID-19, indicating the need to administer remdesivir and dexamethasone from the outset. However, remdesivir was not easily available between November 2022 and February 2023.

There are several reports on COVID-19 patients with a history of cerebrovascular disease. It was reported that patients with pre-existing cardiovascular disease were more likely to develop multi-organ dysfunction, deteriorate to a critical condition, and yield poorer clinical outcomes. Concerning therapeutics, a greater proportion of patients with pre-existing cardiovascular disease required mechanical ventilation, higher-order antibiotics, and drugs targeting underlying diseases and complications. In the multivariable analysis, pre-existing cardiovascular disease was significantly associated with a poor clinical outcome [32]. In addition, it was reported that patients with previous cardiovascular disease had a higher risk of severe COVID-19. This association was also observed in clusters of studies that defined the severe manifestation of the disease by clinical parameters, the necessity of intensive care, and in-hospital death. Then, it was suggested that a history of cardiovascular disease might constitute an important risk factor for an unfavorable clinical course of COVID-19, suggesting a need for tailored infection prevention and clinical management strategies for this population at risk [33].

We retrospectively studied patients infected with the SARS-CoV-2 omicron variant who were hospitalized for stroke at a single hospital. Although the clinical features of COVID-19 during hospitalization following a stroke have rarely been reported, the small sample size in this study might contribute to the non-significant results for events that previous studies have identified as significant. Further studies are necessary to determine the complications associated with COVID-19 following a stroke and develop appropriate therapeutic strategies for the same.

## Conclusions

Per our study, hospitalized stroke patients who contract COVID-19 caused by the SARS-CoV-2 omicron variant are characterized by a higher mortality rate. We also observed a correlation between high mRS scores and high NIHSS scores at the time of COVID-19 onset and disease severity. In addition, the D-dimer levels in patients with mild COVID-19 are significantly lower than in deceased patients. A COVID-19-induced coagulopathy can lead to myocardial and cerebral infarctions following an ischemic stroke, resulting in mortality. Therefore, anticoagulant therapy should be seriously considered for such patients.
